# Immune Cell Profiles of Patients with Sickle Cell Disease during Parvovirus B19–Induced Transient Red Cell Aplasia

**DOI:** 10.3390/vaccines12090984

**Published:** 2024-08-29

**Authors:** E. Kaitlynn Allen, Rhiannon R. Penkert, Jane S. Hankins, Sherri L. Surman, Lee-Ann Van de Velde, Alyssa Cotton, Randall T. Hayden, Li Tang, Xiaomeng Yuan, Ying Zheng, Paul G. Thomas, Julia L. Hurwitz

**Affiliations:** 1 Department of Immunology, St. Jude Children’s Research Hospital, Memphis, TN 38105, USA; emma.allen@stjude.org (E.K.A.); lee-ann.vandevelde@stjude.org (L.-A.V.d.V.); paul.thomas@stjude.org (P.G.T.); 2 Department of Infectious Diseases, St. Jude Children’s Research Hospital, Memphis, TN 38105, USA; rpenkert@uoregon.edu (R.R.P.); sherri.surman@stjude.org (S.L.S.); 3 Department of Hematology, St. Jude Children’s Research Hospital, Memphis, TN 38105, USA; jane.hankins@stjude.org (J.S.H.); alyssa.cotton@stjude.org (A.C.); 4 Department of Global Pediatric Medicine, St. Jude Children’s Research Hospital, Memphis, TN 38105, USA; 5 Department of Pathology, St. Jude Children’s Research Hospital, Memphis, TN 38105, USA; randall.hayden@stjude.org; 6 Department of Biostatistics, St. Jude Children’s Research Hospital, Memphis, TN 38105, USA; li.tang@stjude.org (L.T.); xiaomeng.yuan@stjude.org (X.Y.); ying.zheng@stjude.org (Y.Z.); 7 Department of Microbiology, Immunology and Biochemistry, University of Tennessee Health Science Center, Memphis, TN 38163, USA

**Keywords:** sickle cell disease, parvovirus B19 infection, transient red cell aplasia, aberrant immune phenotypes, nasal wash cytokines

## Abstract

Parvovirus B19 frequently infects children and targets cells of the erythroid lineage. Although healthy children rarely suffer severe disease, children with sickle cell disease (SCD) can experience transient red cell aplasia (TRCA), hospitalization, and life-threatening anemia upon first virus exposure. Given that children with SCD can also suffer chronic inflammation and that parvovirus B19 has been associated with autoimmune disease in other patient populations, we asked if parvovirus B19 infections contributed to acute and chronic immune abnormalities in children with SCD. Nineteen hospitalized patients with SCD and parvovirus B19–induced TRCA were evaluated. Blood tests included CBC, flow cytometry, and total antibody isotype analyses. Cytokine/chemokine analyses were performed on nasal wash (NW) samples, representing a common site of viral entry. Unusually high white blood cell count (WBC) and absolute neutrophil count (ANC) values were observed in some patients. A correlation matrix with Day 0 values from the 19 patients then identified two mutually exclusive phenotype clusters. Cluster 1 included WBC, ANC, absolute reticulocyte count (ARC), absolute lymphocyte count (ALC), lactate dehydrogenase (LDH), NW cytokines/chemokines, % naïve cells among B cell and T cell populations, and parvovirus-specific IgG. This cluster was negatively associated with virus load, suggesting a signature of successful adaptive immunity and virus control. Cluster 2 included virus load, % CD38^+^CD24^−^ cells among CD19^+^ B cells (termed ‘plasmablasts’ for simplicity), % HLA-DR^low^ cells among CD19^+^ B cells, IgG4, and % memory phenotypes among B cell and T cell populations. Plasmablast percentages correlated negatively with parvovirus-specific IgG, possibly reflecting a non-specific trigger of cell activation. All patients were released from the hospital within 1 week after admission, and the highest WBC and ANC values were eventually reduced. Nonetheless, a concern remained that the acutely abnormal immune profiles caused by parvovirus B19 infections could exacerbate chronic inflammation in some patients. To avoid the numerous sequelae known to affect patients with SCD following hospitalizations with parvovirus B19, rapid development of a parvovirus B19 vaccine is warranted.

## 1. Introduction

Parvovirus B19 is typically transmitted between humans by aerosol inhalation. The virus has tropism for erythroid progenitor cells and causes erythroblast hypoplasia. While healthy children usually suffer limited disease upon infection with parvovirus B19 [1], children with sickle cell disease (SCD) can suffer life-threatening consequences. Unlike their healthy counterparts, children with SCD have inherited red blood cell disorders. Increased demands for red cell production render these patients vulnerable to transient red cell aplasia (TRCA) [2,3,4] upon first parvovirus B19 exposures.

Patients who suffer parvovirus B19 infections and TRCA will usually recover rapidly after mounting a protective, virus-specific immune response [1]. Nonetheless, the virus can cause debilitating acute vaso-occlusive crises. TRCA can cause severe sequelae in patients with SCD, including myocarditis, fatal bone marrow embolism, glomerulonephritis, and stroke-induced chronic neurological deficits [5,6,7,8].

Children with SCD can also suffer from inflammatory disease and in other populations, parvovirus B19 associates with autoimmune diseases such as lupus [9]. We therefore asked if children with SCD who are hospitalized with parvovirus B19 infections experience both acute and chronic immune abnormalities. To address this question, we queried associations between parvovirus B19, virus-specific IgG [10,11], blood cell immunophenotypes, total serum immunoglobulin isotypes, and cytokines/chemokines in nasal washes (NWs).

## 2. Materials and Methods

### 2.1. Participant Selection and Data Collection

We previously performed a single-institution iSCREEN study (NCT02261480) that enrolled 20 children with SCD during acute parvovirus B19 infections [10,11] and collected samples on Days 0 (baseline, the day of hospitalization), 7, 30, and 120. Children had a diagnosis of SCD of any genotype, were older than 1 year of age, and were admitted to St. Jude Children’s Research Hospital (St. Jude) upon diagnosis of parvovirus B19–induced TRCA (defined as worsened anemia with insufficient compensatory reticulocytosis and febrile illness). Parvovirus B19 infection was revealed by a positive diagnostic virus-specific IgM ELISA or PCR. The diagnostic test was performed on Days 0 and 30 by Quest Diagnostics (Secaucus, NJ, USA) or ARUP Laboratories (Salt Lake City, UT, USA) using a commercial kit (B19V-specific EIAs for B19V-specific IgM and IgG (Biotrin International, Dublin, Ireland)). One value was missing for the test on Day 30. Exclusion criteria included receipt of chronic erythrocyte transfusion therapy and/or current epistaxis. The majority of patients were on hydroxyurea therapy at the time of hospitalization. All patients in this study were discharged from the hospital within a 1-week time frame. This study was approved by the St. Jude Institutional Review Board (IRB, 24 September 2014) with a consent for participation signed by all legal guardians.

Nineteen of the twenty patients had residual samples available for flow cytometry on Day 0 and were therefore further tested for phenotype correlations. Additional blood tests included complete blood counts (CBCs), virus loads, and total serum immunoglobulin isotypes. Absolute reticulocyte counts (ARC) and lactate dehydrogenase (LDH) were measured in a CLIA-certified laboratory at St. Jude. ARC measurements were on an automated hematology analyzer. LDH was measured with an LDH ver.2 assay and a Roche Cobas 6000 c501 analyzer. Parvovirus B19 was measured with a quantitative real-time PCR assay performed at Quest Laboratories. Virus measurements were available for 16 patients on Day 0, 19 patients on Day 7, 19 patients on Day 30, and 18 patients on Day 120. The lower limit of detection was 100 and the upper limit of detection was 100,000,000 copies/mL.

Control patients were male and female children between the ages of 5 and 13 years. Controls had SCD, but had not experienced a recent hospitalization due to parvovirus B19 (TBANK, Pro00000728, St. Jude IRB-approved 8 September 2009). There were two control groups, one of which was known to have had a previous exposure to parvovirus B19.

### 2.2. Total Serum Immunoglobulin Measurements

Total immunoglobulin isotypes in sera were quantified with a bead-based multiplex immunoassay (Millipore Sigma, Billerica, MA, USA) using a Luminex 200 Multiplexing Instrument with xPONENT software (Luminex, Austin, TX, USA; https://int.diasorin.com) and analyzed using Milliplex Analyst software (Millipore Sigma, Billerica, MA, USA; https://www.emdmillipore.com). The tested isotypes included IgM, IgG1, IgG2, IgG3, IgG4, and IgA.

### 2.3. Cytokine/Chemokine Measurements

Measurements of 38 cytokines/chemokines in NW on Day 0 were accomplished using a Multiplex (Millipore MAP Kit cat #HCYTMAG-60K-PX38, Millipore Sigma, Burlington, MA, USA) and a Luminex 200 Multiplexing Instrument, as above. Milliplex Analyst software was used for data analyses, as above. Cytokines/chemokines included EGF, eotaxin, FGF-2, FKN, Flt-3L, G-CSF, GM-CSF, GRO, IFNa2, IFNγ, IL-1α, IL-1β, IL-1RA, IL-2, IL-3, IL-4, IL-5, IL-6, IL-7, IL-8, IL-9, IL-10, IL12-p40, IL12-p70, IL-13, IL-15, IL-17a, IP-10, MCP-1, MCP-3, MDC, MIP-1α, MIP-1β, sCD40L, TGFα, TNFα, TNFβ, and VEGF. There were values available for 14 of the 19 patients. Measurements (pg/mL) were often below detection. When factors gave scores at the lower or upper limit of detection (LLD or ULD, respectively), those scores were used for statistical analyses. Serum cytokines for this patient population were previously described [10].

### 2.4. Flow Cytometry

Peripheral blood mononuclear cells (PBMC) were isolated at baseline and stored in liquid nitrogen. Samples were thawed rapidly at 37 °C and transferred to a sterile 15 mL tube. Warmed complete-RPMI was added dropwise to a final volume of 10 mL and cells were centrifuged at 500× *g* for 5 min. Cells were quantified using a Vi-Cell Counter (Beckman Coulter, Brea, CA, USA) and resuspended in FACS Buffer (PBS containing 0.5% fetal bovine serum [FBS] and 2 mM EDTA) for cytometric analyses. Cells were treated with 1:100 of human Fc receptor blocking solution (BioLegend, San Diego, CA, USA, cat# 422302) for 10 min at 4 °C, prior to surface staining. Staining was for 30 min at 4 °C in FACS buffer. Tubes were protected from light. Viability dye and antibodies were used at final 1:400 and 1:100 dilutions, respectively.

For B cell staining, the antibody cocktail included viability dye (Ghost Dye Violet 510, cat# 13-0870-T100, Tonbo biosciences, San Diego, CA, USA), anti-CD235a PerCP/Cy5.5 (clone HI264, BioLegend cat# 349110), anti-CD14 PE/Cy7 (clone M5E2, BioLegend cat# 301814), anti-CD19 PE (clone HIB19, BioLegend cat# 982402), anti-IgD BV711 (clone IA6-2, BD cat# 740794), anti-CD27 BV785 (clone O323, BioLegend cat# 302832), anti-CD38 FITC (clone HIT2, BioLegend cat# 303504), anti-CD24 PE/Dazzle594 (clone ML5, BioLegend cat# 311134), anti-HLA-DR BV605 (clone L243, BioLegend cat# 307640), and anti-CD20 AF700 (clone 2H7, BioLegend cat# 302322).

For T cell staining, the antibody cocktail included viability dye (as above), anti-CD3 AF700 (clone UCHT1 Biolegend cat# 300424), anti-CD4 BV421 (clone RPA-T4, BioLegend cat# 300532), anti-CD8 BV785 (clone RPA-T8, BioLegend cat# 301045), anti-CCR7 FITC (clone G043H7, BioLegend cat# 353216), and anti-CD45RO BV605 (clone UCHL1, BioLegend cat# 304238).

For CD56 staining, the antibody cocktail included viability dye (as above), anti-CD56 PE/Dazzle594 (clone HCD56, BioLegend cat# 318348), anti-CD20 PerCP/Cy5.5 (clone 2H7, BioLegend cat# 302326), anti-CD3 PerCP/Cy5.5 (clone OKT3, BioLegend cat# 317336), anti-CD235a PerCP/Cy5.5 (clone HI264, BioLegend cat# 349110), and anti-HLA-DR BV605 (clone L243, BioLegend cat# 307640).

After staining, cells were washed twice and resuspended in FACS buffer. Data were acquired on a BD LSR Fortessa X-20 and analyzed in FlowJo v10 (https://www.flowjo.com).

For B cell analyses, cells were first gated for singlet lymphocytes using FSC/SSC profiles and exclusion of dead cells permeable to the viability dye. Cells were further gated to exclude CD235a^+^ (erythroid) and CD14^+^ (myeloid) cells. Then, among CD19^+^ B cells, percentages were determined for CD38^+^CD24^−^ cells (termed ‘plasmablasts’ for simplicity), IgD^+^CD27^−^ cells (termed ‘naïve cells’), CD27^+^IgD^−^ cells, and HLA-DR^Low^ cells. Among CD235a^−^CD14^−^CD19^−^CD20^−^ cells, CD38^+^ cell percentages were determined.

For T cell analyses, cells were gated using FSC/SSC to define singlet lymphocytes. Dead cells were excluded and CD3^+^ cells were positively gated. For CD3^+^ cells, percentages of CD4^+^CD8^−^ (CD4^+^) or CD8^+^CD4^−^ (CD8^+^) T cells were measured. For the individual CD3^+^CD4^+^ or CD3^+^CD8^+^ T cell populations, percentages of CCR7^+^CD45R0^−^ cells (termed ‘naïve’), CCR7^+^CD45R0^+^ cells (termed ‘T central memory’ (TCM)), and CCR7^−^CD45R0^+^ cells (termed ‘T effector memory’ (TEM)) were measured.

For CD56^+^ cell analyses, cells were gated using FSC/SSC to select singlet lymphoid and myeloid cells. Dead cells were excluded. Percentages of CD56^+^ cells were measured among CD235a^−^CD20^−^HLA-DR^−^CD3^−^ cells.

### 2.5. Statistical Analyses

Spearman’s rank correlation coefficients and *p*-values were computed using both GraphPad Prism software (Boston, MA, USA, version 10.2, www.graphpad.com) and the rcorr function in the R package (version 4.3.0) Hmisc (version 5.1.1). Results were not adjusted for multiple comparisons. Missing values were handled by excluding pairs of variables that contained missing values during the correlation computation. The correlation matrix was generated utilizing the rcorr function in the Hmisc package and visualized through the corrplot function in the corrplot package (version 0.92).

## 3. Results

### 3.1. Cases of Elevated WBC and ANC upon Hospitalization of Patients with SCD and Parvovirus B19 Infections

Our previous iSCREEN study (NCT02261480) examined 20 patients with SCD (median age 7 years, range 4.5 to 14.3 years, 15 HbSS, 3 HbSC, 1 HbSD, 1 HbSβeta^+^thalassemia) who were hospitalized with TRCA due to parvovirus B19 infections [10,11]. Patients recovered quickly and were released from the hospital within 1 week of admission. Among the 20 patients, there were 19 for whom Day 0 PBMC samples were available for additional testing; there was an insufficient sample from one HbSC patient. With the 19 samples, we examined the associations between virus infections and immunophenotypes.

**Figure 1:**
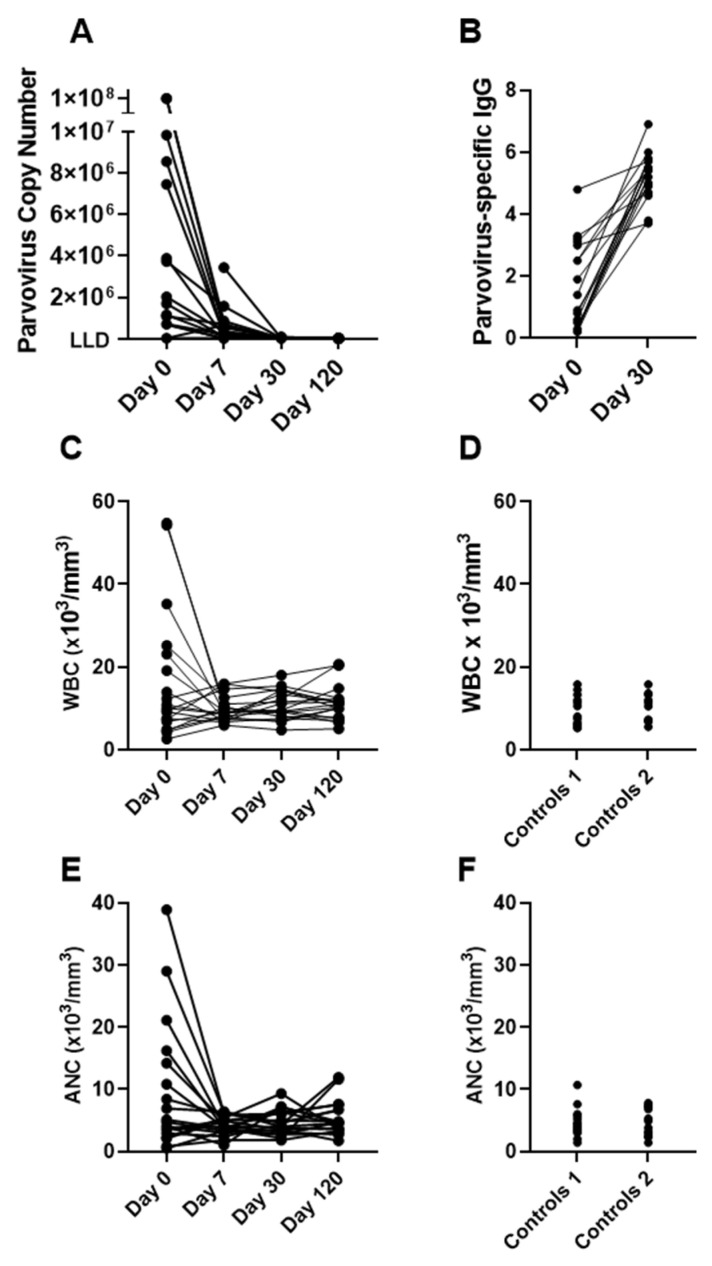
High WBC and ANC on Day 0 in some patients. Patient data (**A**–**C**,**E**) and control data (**D**,**F**) are shown. Controls were individuals with SCD who were not hospitalized with a parvovirus B19 infection. They either had a previous exposure to parvovirus B19 (Controls 1) or were not known to have had a previous exposure to parvovirus B19 (Controls 2). Parvovirus loads and virus-specific IgG (**A**,**B**) have been previously described. WBC and ANC are shown for patients and controls (**C**–**F**).

As previously described, the virus was quickly controlled after hospitalizations as the virus-specific IgG response was mounted (Figure 1A,B). CBC measurements revealed unusually high WBC and ANC among several patients on Day 0, the day of hospitalization. WBC scored higher than 50 × 10^3^/mm^3^ and ANC scored higher than 30 × 10^3^/mm^3^ in one patient, unlike the controls (Figure 1C–F). The controls included patients with SCD who were exposed to parvovirus B19 in the past (Controls 1) or who had no known previous exposure (Controls 2).

To dissect the effects of parvovirus B19 among patients with SCD and to understand aspects of the host response that may be co-regulated and therefore important to the infectious disease outcome, we examined correlations among Day 0 virus loads, CBC numbers, cell phenotypes (focused on B cell and T cell populations), and disease symptoms (e.g., elevated blood cell numbers/percentages, LDH, and nasal wash cytokines (the latter marking a common site of viral entry)).

### 3.2. Two Mutually Exclusive Phenotype Clusters on Day 0

**Figure 2:**
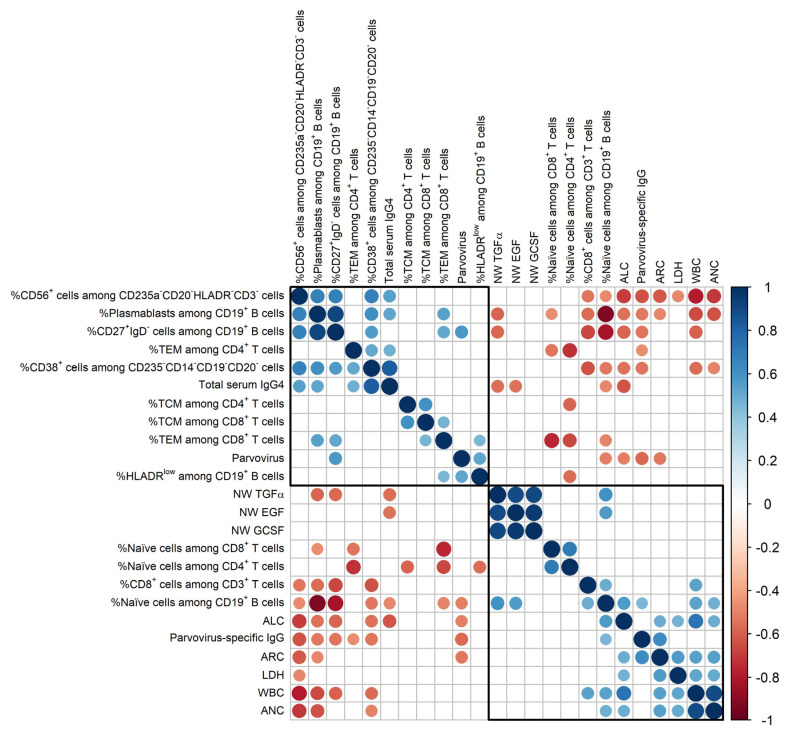
Correlation plot of baseline parameters (Day 0). Nonsignificant (*p* > 0.05) coefficients were not displayed. The sizes of the circles represent the absolute values of the corresponding correlation coefficients, and the colors of the circles indicate the values of the correlation coefficients. The arrangement of parameters was determined using hierarchical clustering (‘hclust’). Cluster 1 and Cluster 2 are defined by the dark black boxes positioned in bottom right and top left corners, respectively.

A correlation matrix with Day 0 data revealed two mutually exclusive phenotype clusters. Dark black boxes in Figure 2 outline Cluster 1 (bottom right) and Cluster 2 (top left). The Day 0 patient age was tested against all variables in Figure 2 but revealed no significant correlations.

Cluster 1 included WBC, ANC, and parvovirus-specific IgG. The negative correlation between parvovirus-specific IgG and virus signified a functional, protective adaptive immune response.

**Figure 3:**
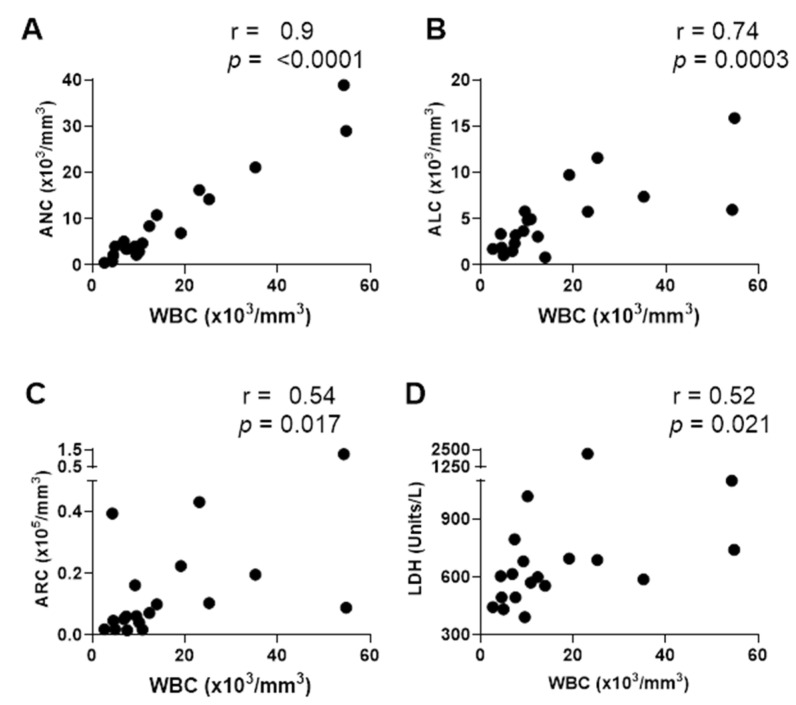
WBC associates positively with other blood phenotypes. Detailed correlations are shown between WBC and ANC (**A**), ALC (**B**), ARC (**C**), and LDH (**D**).

Additional blood phenotypes within Cluster 1 were % naïve cells among lymphocyte subsets, % CD8^+^ cells among CD3^+^ cells, absolute lymphocyte counts (ALC), absolute reticulocyte counts (ARC), and LDH. Detailed illustrations of positive correlations within Cluster 1 are shown in Figure 3.

As shown, WBC correlated positively with ANC (r = 0.90, *p *= <0.0001), ALC (r = 0.74, *p *= 0.0003), ARC (r = 0.54, *p *= 0.017), and LDH (r = 0.52, *p *= 0.021). WBC also correlated positively with % naïve cells among CD19^+^ B cells (r = 0.54, *p *= 0.018), and % CD8^+^ cells among CD3^+^ T cells (r = 0.54, *p *= 0.017).

Apart from blood cell testing, NW samples were tested for 38 cytokines/chemokines. Fourteen of nineteen NW samples were available for analyses. There were a range of values among patients (e.g., EGF values ranged from the LLD to >1000 pg/mL; TGFα values ranged from the LLD to 204 pg/mL). Three representative factors, TGFα, EGF, and GCSF, shown in Figure 2, were associated with Cluster 1. Significant positive associations were observed among these factors with each other. For TGFα and EGF, there were also positive associations with the % naïve cells among CD19^+^ B cells (Figure 2). Additional positive associations with % naïve cells among CD19^+^ B cells included FGF-2, eotaxin, GM-CSF, FKN, IFNα2, IFNγ, IL-10, MCP-3, IL-12p40, IL-12p70, sCD40L, IL-1α, IL-1β, IL-6, IL-8, MIP-1α, MIP-1β, TNFα, and VEGF.

Cluster 2 included % CD38^+^CD24^−^ cells (termed ‘plasmablasts’ for simplicity) among CD19^+^ B cells. Sample plasmablast staining is shown in Supplementary Figure S1 for patients with high and low percentages (Supplementary Figure S1A,B and Supplementary Figure S1C,D, respectively). The majority of plasmablasts (>80% on average among patients) were negative for CD20, as would be expected [12].

**Figure 4:**
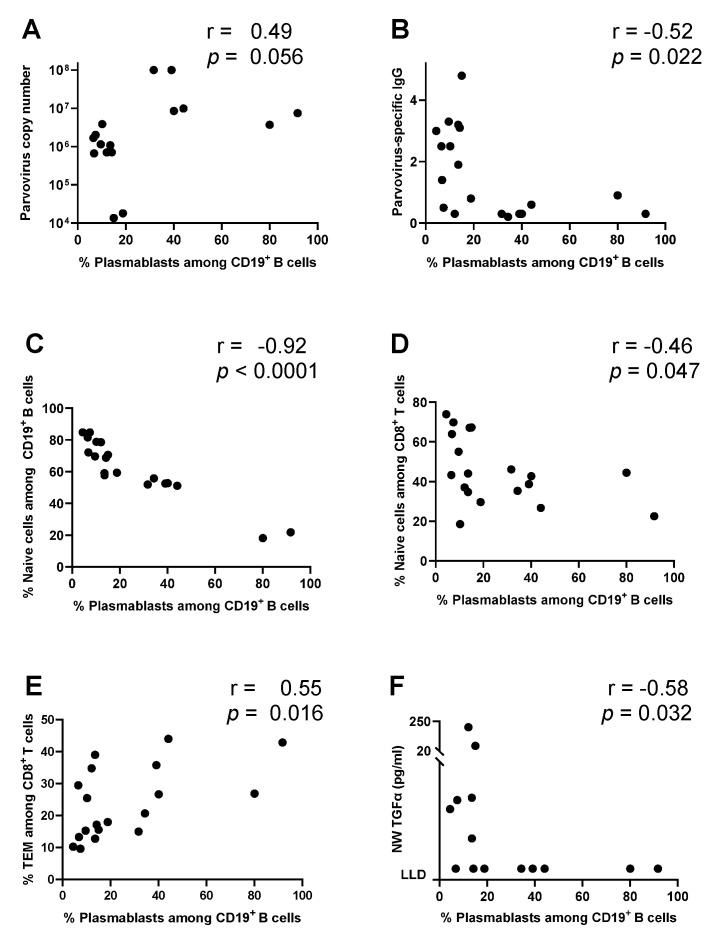
% plasmablasts among CD19^+^ B cells correlate negatively with parvovirus-specific IgG. Associations between % plasmablasts among CD19^+^ B cells and parvovirus copy number (**A**), parvovirus-specific IgG (**B**), % naïve cells among CD19^+^ B cells (**C**), % naïve cells among CD8^+^ T cells (**D**), % TEM among CD8^+^ T cells (**E**), and NW TGFα (**F**) are shown.

Detailed correlations involving % plasmablasts among CD19^+^ B cells are shown in Figure 4. There was a trend toward a positive correlation with virus (r = 0.49, *p *= 0.056), and there was a negative association with parvovirus-specific IgG (r = −0.52, *p *= 0.022, Figure 4A,B). These features suggested that plasmablasts may have been activated, at least in part, by an antigen non-specific trigger [13] and were relatively ineffective at clearing virus.

% plasmablasts among CD19^+^ B cells correlated negatively with % naïve B cells among CD19^+^ B cells and % naïve T cells among CD8^+^ T cells (Figure 4C,D). A positive correlation was observed between % plasmablasts among CD19^+^ B cells and % TEM among CD8^+^ T cells (Figure 4E). There were significant negative correlations between % plasmablasts among CD19^+^ B cells and some NW cytokines/chemokines, including TGFα (*r *= −0.58, *p *= 0.032, Figure 4F).

As shown in Figure 2, % plasmablasts among CD19^+^ B cells correlated negatively with both WBC (r = −0.66, *p *= 0.0022) and ANC (r = −0.64, *p *= 0.0032). There was a positive correlation between % plasmablasts among CD19^+^ B cells and CD27^+^IgD^−^ cells among CD19^+^ B cells, the latter of which was positively associated with virus. CD27 is a TNF receptor family member, known as a marker of human memory B cells, activated B cells (ABC) and plasmablasts [12,14]. CD27^+^ plasmablasts are prevalent in the context of virus infection and autoimmune disease [12,14,15,16,17,18]. % plasmablasts among CD19^+^ B cells also correlated positively with % CD56^+^ cells among CD235a^−^CD20^−^HLA^−^DR^−^CD3^−^ cells (*r *= 0.67, *p *= 0.0017). CD56 is a neural cell adhesion molecule associated with natural killer cells and a variety of other cells including plasma cells in the context of infectious, autoimmune, and malignant diseases [19,20,21,22,23,24,25]. % plasmablasts among CD19^+^ B cells correlated positively with %CD38^+^ cells among CD235a^−^CD14^−^CD19^−^CD20^−^ cells, a phenotype inclusive of plasma cells, that was, in turn, negatively associated with parvovirus B19–specific IgG.

Cluster 2 included HLA-DR^Low^ cells among CD19^+^ B cells, another phenotype that was positively associated with virus (r = 0.52, *p *= 0.04, Figure 2). Possibly a reduction in HLA-DR on B cells provided a mechanism of virus escape from the adaptive immune response [26,27].

**Figure 5:**
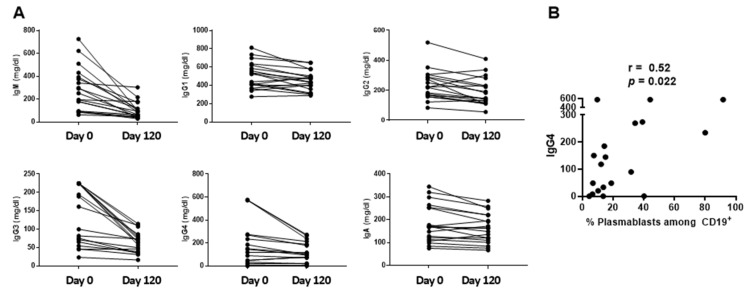
Serum immunoglobulin isotypes. Serum immunoglobulin isotype values are shown for Days 0 and 120 (**A**). A positive correlation was observed between IgG4 (mg/dL) and % plasmablasts among CD19^+^ B cells (**B**).

Total immunoglobulin isotypes in sera were examined on Days 0 and 120 (Figure 5A). The highest values were observed on Day 0. IgG4 was a component of Cluster 2 and was positively associated with % plasmablasts among CD19^+^ B cells (Figure 5B) and % CD38^+^ cells among CD235a^−^CD14^−^CD19^−^CD20^−^ cells (Figure 2). As was the case for WBC and ANC values, the highest total immunoglobulin values (e.g., for IgG4) were ultimately reduced over time (Figure 5A).

## 4. Discussion

A cohort of 19 hospitalized children with SCD suffering from TRCA caused by parvovirus B19 infections were analyzed for immunophenotypes. All patients ultimately mounted an effective parvovirus-specific immune response and controlled the virus, but abnormal immunophenotypes were nonetheless observed. While blood factors have previously been described in patients with SCD (e.g., elevated cytokine/chemokine levels that can be partially controlled by HU [4]), analyses of blood cell phenotypes and NW cytokines have been relatively rare, particularly for patients with SCD and infections with parvovirus B19 [4,28,29,30].

### 4.1. Immunophenotypes on Day 0

A correlation matrix revealed two mutually exclusive phenotype clusters on Day 0. Cluster 1 variables included WBC, ANC, ALC, ARC, LDH, naïve cells among CD19^+^ B cells, naïve cells among CD3^+^ T cells, and parvovirus B19-specific IgG. Variables in Cluster 1 were negatively associated with virus. While Cluster 1 phenotypes were associated with adaptive immunity and virus control, they may have also contributed to disease. High WBC levels may represent a consequence of, but also a contributor to, TRCA-associated vaso-occlusion, ischemia, and tissue damage [28,31,32,33,34,35,36].

In Cluster 2, there were non-naïve B cell and T cell populations including plasmablasts and memory cells. The finding that % plasmablasts among CD19^+^ B cells correlated negatively with parvovirus-specific IgG suggested that cells may have been triggered, at least in part, by an antigen non-specific trigger and were relatively poor at virus control [13,14,16,37,38,39]. Non-specific, polyclonal activation, if present, may have hampered adaptive immunity and promoted virus escape. B cells with low HLA-DR expression [26,27,40], another phenotype associated with Cluster 2, may have additionally assisted virus escape from adaptive immunity. Yet another factor, serum IL-10 (previously described [10]) could have hampered immune protection.

It is possible that Cluster 2 variables represented an earlier time point after virus exposure compared to Cluster 1 variables, prior to the mounting of a successful adaptive immune response. Of note, adaptive immune responses are generally successful in patients with SCD after parvovirus B19 infections; the pathogen is usually cleared and patients are usually protected against future parvovirus B19 exposures [4]. Numerous other factors may have contributed to the presence of Cluster 1 versus Cluster 2 phenotypes among patients with SCD, including drug treatments [4], genetic backgrounds, and environmental influences.

CD56 and IgG4 were additional phenotypes associated with Cluster 2. These can each mark serious conditions. CD56 is an NK marker, but also marks plasma cells in the context of infectious, autoimmune, and malignant diseases [19,20,21,22,23,24,25]. High serum IgG4 is reminiscent of IgG4-related disease (RD) [20,21,22,24,25,41,42,43]. IgG4-RD is a multi-organ inflammatory disease in which hypergammaglobulinemia and circulating plasmablasts/plasma cells are prevalent [41,43,44,45,46,47]. Manifestations of disease include autoimmune pancreatitis, sinusitis, and thyroiditis [43]. If parvovirus B19 infection can initiate the non-specific stimulation of B cells and/or antigen mimicry, an autoreactive immune response may ensue [48,49].

### 4.2. Might Acute Immune Abnormalities Following Parvovirus B19 Infections Exacerbate Chronic Inflammatory Disease in Patients with SCD?

While all patients in this study gained control over parvovirus B19 and there was a reduction in the highest values for WBC, ANC, and IgG4 over time, the long-term consequences of acute immune abnormalities remain unknown. Patients with SCD often suffer chronic inflammation [49,50,51,52], a condition that may be falsely attributed solely to the red cell defect. Associations between parvovirus B19 and autoimmune diseases have been described in other patient populations [37,48,49,51,52,53,54,55,56,57,58,59,60]. For example, rheumatoid arthritis is frequently observed among adults suffering from parvovirus B19 infections. Arthropathy can occur in more than 50% of parvovirus B19–infected individuals, with presentations including arthritis in the hands, wrists, knees, and ankles. Systemic lupus erythematosus is similarly associated with parvovirus B19 infections [9], as are other conditions of immune dysfunction [37,48,49,51,52,53,54,55,56,57,58,59,60]. The acute abnormalities described here add to numerous sequelae already known to follow parvovirus B19 infections in children with SCD and highlight an urgent need to improve therapies and develop a pediatric vaccine [11].

### 4.3. Study Limitations

Our study included a small population of 19 SCD patients, all from one institution. The follow-up period was 120 days and sequelae may have taken longer to observe. Analyses were not controlled for multiple comparisons or potential confounders, such as possible co-infections or variant treatment regimens. Certain assays involved upper and lower limits of detection, beyond which patient differences could not be determined. Due to these limitations, the performance of larger studies is encouraged to confirm and expand upon current findings.

### 4.4. Summary

We describe immune profiles associated with TRCA following parvovirus B19 infections in children with SCD. A correlation matrix revealed two mutually exclusive clusters of positively associated variables on Day 0. Cluster 1 included WBC ANC, ALC, ARC, LDH, % naïve B cells among CD19^+^ B cells, % naïve cells among CD8^+^ T cells, and NW cytokines. Cluster 2 included parvovirus B19 copy number, % plasmablasts among CD19^+^ B cells, IgG4, HLA-DR^Low^ cells among CD19^+^ B cells, and various memory cell phenotypes. Inverse correlations between immune cells in Cluster 2 and parvovirus B19–specific IgG suggested that an antigen non-specific signal may have triggered polyclonal cell activation and may have hampered virus control. Low HLA-DR expression on B cells was another phenotype in Cluster 2 that may have impeded virus-specific immunity. While hospitalizations were brief for all patients and robust virus-specific immunity was eventually mounted, a question remained concerning the impact of acute immune abnormalities on chronic inflammation. To avoid sequelae in patients with SCD after parvovirus B19 exposures, extended research and rapid development of a parvovirus B19 vaccine are encouraged.

## Data Availability

Data are available upon request to the corresponding author.

## Supplementary Materials

The following supporting information can be downloaded at: https://www.mdpi.com/article/10.3390/vaccines12090984/s1, Supplementary Figure S1: CD19^+^ B cell analyses. Flow cytometry profiles are shown for two patients with different % plasmablast percentages among CD19^+^ B cells. Data from the two patients are in (A,B) and (C,D), respectively. For each patient, CD19^+^ B cells (shown in histograms (A,C) were selected and analyzed for CD24 and CD38 markers (shown in (B,D)). The boxed areas in (B,D) are termed ‘plasmablasts’ for simplicity.

## Abbreviations

PBMC, peripheral blood mononuclear cells; ARC, absolute reticulocyte count; ALC, absolute leukocyte count.
