# Supplementary figures and images for: Immune Cell Profiles of Patients with Sickle Cell Disease during Parvovirus B19–Induced Transient Red Cell Aplasia
Source: Vaccines (Basel). 2024 Aug 29;12(9):984. doi: 10.3390/vaccines12090984 (PMC11435734; doi:10.3390/vaccines12090984)

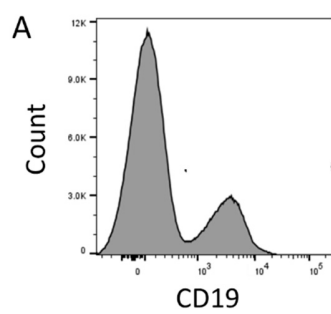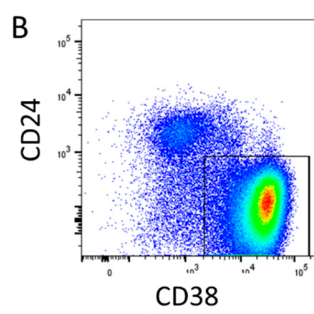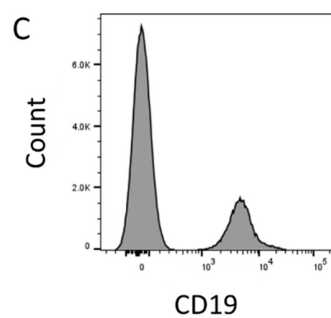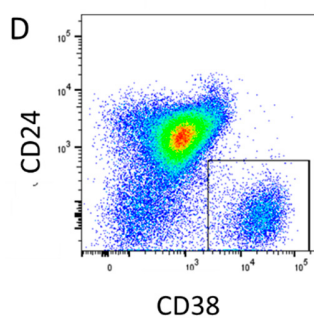

Supplement: Supplementary file 1 [file vaccines-12-00984-s001.zip › vaccines-3074125-supplementary.pdf]
